# Investigating the association between allergen-specific immunoglobulin E, cancer risk and survival

**DOI:** 10.1080/2162402X.2016.1154250

**Published:** 2016-03-28

**Authors:** Wahyu Wulaningsih, Lars Holmberg, Hans Garmo, Sophia N. Karagiannis, Staffan Ahlstedt, Håkan Malmstrom, Mats Lambe, Niklas Hammar, Göran Walldius, Ingmar Jungner, Tony Ng, Mieke Van Hemelrijck

**Affiliations:** a Cancer Epidemiology Group, Division of Cancer Studies, King's College London, London, UK; b Department of Surgical Sciences, Uppsala University Hospital, Uppsala, Sweden; c Regional Cancer Centre, Uppsala, Sweden; d St. John's Institute of Dermatology, Division of Genetics and Molecular Medicine, Faculty of Life Sciences and Medicine, King's College London, NIHR Biomedical Research Centre at Guy's and St. Thomas's Hospitals and King's College London, London, UK; e Center of Allergy Research, Institute of Environmental Medicine, Karolinska Insitutet, Stockholm, Sweden; f Unit of Epidemiology, Institute of Environmental Medicine, Karolinska Institutet, Stockholm, Sweden; g Department of Medical Epidemiology and Biostatistics, Karolinska Institutet, Stockholm, Sweden; h AstraZeneca R&D, Mölndal, Sweden; i Unit of Cardiovascular Epidemiology, Institute of Environmental Medicine, Karolinska Institutet, Stockholm, Sweden; j Department of Medicine, Clinical Epidemiological Unit, Karolinska Institutet and CALAB Research, Stockholm, Sweden; k Richard Dimbleby Department of Cancer Research, Randall Division and Division of Cancer Studies, King's College London, London, UK; l Division of Hematology/Oncology, Faculty of Medicine, Gadjah Mada University, Yogyakarta, Indonesia

**Keywords:** Allergy, atopy, cancer, immunoglobulin E, cohort

## Abstract

Prior findings linking allergy and cancer have been inconsistent, which may be driven by diverse assessment methods. We used serum specific immunoglobulin E (IgE) against common inhalant allergens that was assessed prior to cancer diagnosis in studying this association. We selected 8,727 Swedish men and women who had measurements of serum allergen-specific IgE and total IgE between 1992 and 1996. Multivariable Cox regression using age as a timescale was performed to assess the associations of IgE sensitization, defined by any levels of serum specific IgE ≥35 kU/L, with risk of overall and specific cancers. A test for trend was performed by assigning scores derived from allergen-specific IgE levels at baseline as an ordinal scale. Kaplan–Meier curves and log-rank test were used to assess cancer survival by IgE sensitization status. During a mean follow-up of 16 year, 689 persons were diagnosed with cancer. We found an inverse association between IgE sensitization and cancer risk, with a hazard ratio (HR) of 0.83 and 95% confidence intervals (CI) of 0.70–0.99. A similar trend was seen with specific IgE scores overall (P_trend_ = 0.007) and in women (P_trend_ = 0.01). Although IgE sensitization was not associated with risk of common site-specific cancers, serum specific IgE scores were inversely associated with melanoma risk in men and women combined, and with risk of female breast and gynecological cancers combined. No association with survival was observed. The association between circulating IgE levels and incident cancer may point toward a role of T-helper 2 (T_H_2)-biased response in development of some cancers.

## Introduction

Allergy is a hypersensitivity reaction initiated by specific immunologic mechanisms.^1^ Accruing evidence has indicated that allergy may be associated with the development of cancer, and is largely divided into two opposing views: (1) allergy may reduce cancer risk and (2) it may increase cancer risk.^2^ The first may be explained by the immunosurveillance hypothesis, which states that increased immune surveillance following hyper-reactive immune responses may hinder the development of cancer.^2^ Similarly, the prophylaxis hypothesis suggests that physical effects of allergy symptoms may prevent cancer by removal of potential carcinogens.^3^ The opposing hypotheses include a shift in T-helper balance, which determines the type of immune responses elicited. Predominance of T_H_2 over T_H_1 underlies the hypersensitivity reactions in allergy, and is thought to divert immune responses from the tumor-eradicating T_H_1 counterpart.^4^ Additionally, allergic inflammation may lead to initiation and promotion of cancer directly or through indirect mechanisms.^5^

Observational findings linking allergy and cancer are inconclusive.^6^ A crude way to determine presence of allergy would be using total IgE levels in the serum that may reflect the imbalance in the immune system, but without knowing its specificity makes the association between allergy and cancer challenging.^7^ Atopy, which refers to the genetic pre-disposition of developing IgE-mediated hypersensitivity or IgE sensitization against allergens,^1^ is often evaluated to unpick the role of allergy. Most previous studies crudely classified individuals into “atopic” and “non-atopic” based on self-reported history,^8-10^ which relies on an individual's recall and may incur a loss of time-specific information. Other studies defined atopy based on laboratory evidence, such as the presence of circulating IgE.^1^ The use of serum IgE against specific allergen provides more insight into how IgE sensitization may be associated to cancer, but may be hampered by the use of imprecise and/or different assessment methods. Findings based on pre-cancer diagnostic levels of allergen-specific IgE are limited and no studies have investigated the impact on cancer survival of pre-diagnostic allergen-specific IgE.^11-13^

In the Swedish Apolipoprotein Mortality Risk Study (AMORIS), we previously investigated the association between total IgE and the risk of cancer in 24,820 individuals. A weak inverse association was found albeit not statistically significant, and results were similar when data from our cohort was combined in a meta-analysis with previous findings.^7^ The recently updated AMORIS database now contains information on serum specific IgE utilizing comparable determinations, more participants, and a longer follow-up (up to 25 y). To gain further insight into the association between allergy and cancer, we now assessed serum specific IgE against common inhalant allergens in relation to risk of developing cancer and death after cancer diagnosis.

## Results

Characteristics of study participants by IgE sensitization status are shown in Table 1. The average age at baseline was 40 y, and over half the study population were female (59%). During follow-up (median: 18.6 y), 689 incident cancer cases were identified. A total of 194 individuals died following cancer diagnosis, among which 146 died from cancer. The most common three types of cancer were prostate, female breast and colorectal cancers. The relative distribution of specific IgE scores to total IgE is shown in Table S1.

**Table 2.** Specific IgE scores in CALAB and corresponding serum concentrations.

Specific IgE score	Serum concentrations (kU/L)	Serum IgE levels
0	<0.35	Absent/undetectable
1	0.35 – 0.70	Low level
2	0.70 – 3.50	Moderate level
3	3.50 – 17.5	High level
4	17.5 – 50	Very high level
5	50 – 100	Very high level
6	≥100	Very high level

**Table 1.** Characteristics of study participants by IgE sensitization status.

	IgE sensitization	
	No (n = 4,714)	Yes (n = 4,013)
Age (years)		
Mean (SD)	42.26 (13.74)	37.31 (12.66)
Sex, male, n (%)	1661 (35.24)	1945 (48.47)
Socioeconomic status, n (%)		
High	1912 (40.56)	1423 (35.46)
Low	2074 (44.00)	1697 (42.49)
Unclassified/missing	728 (15.44)	893 (22.25)
History of chronic respiratory disease, n (%)	67 (1.42)	92 (2.29)
Year of measurement, n (%)		
1992–1994	1192 (25.29)	986 (24.57)
1994–1996	2682 (56.89)	1997 (49.76)
1996	840 (17.82)	1030 (25.67)
Total IgE (kU/L)		
<25	1765 (37.44)	285 (7.10)
25–100	1937 (41.09)	1355 (33.77)
≥100	1012 (21.47)	2373 (59.13)
Mean follow-up in years, Mean (SD)	15.86 (3.59)	16.05 (3.23)
Any cancer during follow-up, n (%)		
All cancer	443 (9.40)	246 (6.13)
Breast (female)	115 (2.44)	50 (1.25)
Prostate	55 (1.17)	42 (1.05)
Colorectal	43 (0.91)	21 (0.52)
Gynecological	41 (0.87)	12 (0.30)
hematological	31 (0.66)	22 (0.55)
Melanoma	27 (0.57)	10 (0.25)
Pulmonary	21 (0.45)	14 (0.35)
Bladder	14 (0.30)	11 (0.27)
NMSC	16 (0.34)	9 (0.22)
CNS	11 (0.23)	9 (0.22)
Kidney	12 (0.25)	9 (0.22)

n = number of participants.

We assessed cancer risk based on IgE sensitization status and specific IgE scores as categories Table 2. When using 0.35 kU/L as the cut-off point for specific IgE, which indicates IgE sensitization, we did not observe any association with risk of cancer (Table 3). However, a statistically significant inverse trend was observed when using serum specific IgE scores in the overall study population (P_trend_ = 0.03). In sex stratification, a similar association was observed in women. Adjustment for serum total IgE showed stronger associations and an inverse association between IgE sensitization and cancer risk in the overall population (HR: 0.83 (95% CI: 0.70–0.99). No association was noted in men and women separately. When stratifying the analyses by serum total IgE levels, the inverse trend was only seen between serum allergen-specific IgE scores and cancer risk in the overall population and in women with total IgE levels >100 kU/L (Table 3). Similar associations were observed when additionally adjusting our model for the number of allergen tested, the number of positive results, or the ratio between the two (results not shown).

**Table 3.** Associations between IgE sensitization, serum specific IgE scores and risk of incident cancer overall and by sex with chronological age as timescale.

		HR (95% CI)						
		IgE sensitization		Specific IgE scores^§^				
	n cancer/n total	No	Yes	0	1–2	3–4	5–6	P_trend_
Both men and women								
n		4714	4013	4714	6402	8331	8727	
Multivariable model	689/8727	1.0 (Ref)	0.88 (0.75–1.03)	1.0 (Ref)	1.00 (0.82–1.21)	0.74 (0.59–0.93)	0.91 (0.55–1.51)	0.03
Additional adjustment for total IgE	689/8727	1.0 (Ref)	0.83 (0.70–0.99)	1.0 (Ref)	0.95 (0.78–1.16)	0.69 (0.54–0.88)	0.79 (0.47–1.32)	0.007
Stratification by total IgE (kU/L)								
	191/2050	1.0 (Ref)	0.79 (0.48–1.32)	1.0 (Ref)	0.86 (0.51–1.47)	0.48 (0.12–1.99)	N/A	0.29
25–100	238/3292	1.0 (Ref)	0.85 (0.64–1.13)	1.0 (Ref)	0.91 (0.65–1.28)	0.77 (0.51–1.16)	N/A	0.18
≥100	260/3385	1.0 (Ref)	0.82 (0.63–1.06)	1.0 (Ref)	1.00 (0.74–1.34)	0.66 (0.48–0.91)	0.81 (0.47–1.39)	0.02
Men								
n		1661	1945	1661	732	1003	210	
Multivariable model	277/3606	1.0 (Ref)	0.97 (0.76–1.23)	1.0 (Ref)	1.01 (0.75–1.36)	0.89 (0.65–122)	1.27 (0.64–2.52)	0.80
Additional adjustment for total IgE	277/3606	1.0 (Ref)	0.89 (0.69–1.17)	1.0 (Ref)	0.95 (0.69–1.29)	0.83 (0.59–1.16)	1.10 (0.54–2.23)	0.42
Stratification by total IgE (kU/L)								
<25	53/649	1.0 (Ref)	0.71 (0.29–1.76)	1.0 (Ref)	0.59 (0.20–1.78)	1.11 (0.27–4.67)	N/A	0.64
25–100	97/1377	1.0 (Ref)	0.83 (0.54–1.28)	1.0 (Ref)	0.99 (0.59–1.66)	0.68 (0.38–1.23)	N/A	0.24
≥100	127/1580	1.0 (Ref)	0.97 (0.67–1.41)	1.0 (Ref)	1.00 (0.64–1.57)	0.89 (0.57–1.40)	1.23 (0.58–2.61)	0.94
Women								
n		3053	2068	3053	956	926	186	
Multivariable model	412/5121	1.0 (Ref)	0.83 (0.67–1.03)	1.0 (Ref)	0.98 (0.76–1.27)	0.64 (0.45–0.90)	0.72 (0.34–1.53)	0.01
Additional adjustment for total IgE	412/5121	1.0 (Ref)	0.81 (0.64–1.02)	1.0 (Ref)	0.96 (0.74–1.24)	0.61 (0.42–0.87)	0.64 (0.30–1.39)	0.01
Stratification by total IgE (kU/L)								
<25	138/1401	1.0 (Ref)	0.82 (0.44–1.53)	1.0 (Ref)	0.98 (0.53–1.83)	N/A	N/A	0.30
25–100	141/1915	1.0 (Ref)	0.87 (0.59–1.26)	1.0 (Ref)	0.85 (0.54–1.34)	0.91 (0.52–1.58)	N/A	0.51
≥100	133/1805	1.0 (Ref)	0.73 (0.52–1.04)	1.0 (Ref)	0.98 (0.66–1.46)	0.51 (0.31–0.83)	0.63 (0.29–1.39)	0.01

§ Highest specific IgE scores recorded at baseline.

N/A = not applicable; n = number of participants.

All models were adjusted for sex (except for sex-specific analysis), socioeconomic status, period of measurement and history of chronic pulmonary disease.

Similar associations to overall cancers were found when assessing all cancers excluding NMSC (Table 4). No statistically significant association was found between positive IgE sensitization and risk of specific cancer types. When observing trends across allergen-specific IgE scores, we found a lower risk of melanoma with higher specific IgE in both men and women combined (P_trend_ = 0.04). No association was observed for other cancer sites. To further investigate the driver of the inverse association observed in women, we combined cancers of female genital organs (breast and gynecological cancers) as a single outcome and a protective effect of higher specific IgE scores was observed (P_trend_ = 0.04).

**Table 4.** Associations between IgE sensitization, serum specific IgE scores and risk of site-specific cancers by sex with chronological age as timescale.

		HR (95% CI)						
		IgE sensitization		Specific IgE scores^§^				
	n cancer	No	Yes	0	1–2	3–4	5–6	P_trend_
Both men and women								
All excluding NMSC	664	1.0 (Ref)	0.82 (0.69–0.99)	1.0 (Ref)	0.96 (0.78–1.18)	0.67 (0.52–0.85)	0.81 (0.48–1.36)	0.005
Colorectal	64	1.0 (Ref)	0.71 (0.39–1.26)	1.0 (Ref)	0.75 (0.38–1.51)	0.60 (0.27–1.34)	1.23 (0.28–5.42)	0.32
hematological	53	1.0 (Ref)	0.95 (0.51–1.76)	1.0 (Ref)	1.16 (0.58–2.30)	0.66 (0.27–1.59)	1.22 (0.27–5.46)	0.62
Melanoma	37	1.0 (Ref)	0.53 (0.24–1.17)	1.0 (Ref)	0.79 (0.33–1.88)	0.32 (0.09–1.13)	N/A	0.04
Pulmonary	35	1.0 (Ref)	0.87 (0.41–1.84)	1.0 (Ref)	1.24 (0.56–2.76)	0.41 (0.11–1.43)	0.94 (0.12–7.44)	0.33
Bladder	25	1.0 (Ref)	1.20 (0.50–2.87)	1.0 (Ref)	1.45 (0.56–3.77)	0.95 (0.29–3.14)	N/A	0.89
NMSC	25	1.0 (Ref)	0.97 (0.40–2.39)	1.0 (Ref)	0.62 (0.17–2.20)	1.56 (0.55–4.44)	N/A	0.74
Kidney	21	1.0 (Ref)	1.09 (0.40–2.95)	1.0 (Ref)	1.50 (0.52–4.30)	0.27 (0.03–2.23)	3.42 (0.63–18.50)	0.95
CNS	20	1.0 (Ref)	1.33 (0.47–3.73)	1.0 (Ref)	1.82 (0.62–5.33)	0.88 (0.21–3.61)	N/A	0.74
Men								
All excluding NMSC	267	1.0 (Ref)	0.89 (0.68–1.16)	1.0 (Ref)	0.95 (0.69–1.31)	0.80 (0.57–1.13)	1.12 (0.55–2.28)	0.37
Prostate	97	1.0 (Ref)	0.93 (0.38–1.04)	1.0 (Ref)	0.86 (0.50–1.48)	0.93 (0.53–1.63)	2.31 (0.78–6.82)	0.79
Colorectal	35	1.0 (Ref)	0.71 (0.34–1.49)	1.0 (Ref)	0.77 (0.31–1.88)	0.54 (0.19–1.53)	1.83 (0.38–8.77)	0.52
hematological	23	1.0 (Ref)	1.13 (0.44–2.89)	1.0 (Ref)	1.11 (0.36–3.35)	1.12 (0.35–3.62)	1.35 (0.15–12.15)	0.78
Pulmonary	15	1.0 (Ref)	1.07 (0.35–3.26)	1.0 (Ref)	1.41 (0.42–4.71)	0.57 (0.11–3.02)	2.38 (0.24–23.99)	0.93
Melanoma	12	1.0 (Ref)	0.56 (0.15–2.06)	1.0 (Ref)	0.65 (0.13–3.18)	5.31 (0.10–2.77)	N/A	0.31
Women								
All excluding NMSC	397	1.0 (Ref)	0.81 (0.63–1.03)	1.0 (Ref)	0.98 (0.75–1.28)	0.57 (0.39–0.83)	0.65 (0.30–1.42)	0.008
Breast and gynecological	218	1.0 (Ref)	0.76 (0.55–1.06)	1.0 (Ref)	0.85 (0.58–1.24)	0.68 (0.43–1.10)	0.36 (0.09–1.47)	0.04
Breast	165	1.0 (Ref)	0.83 (0.57–1.20)	1.0 (Ref)	0.95 (0.63–1.45)	0.69 (0.40–1.19)	0.49 (0.12–2.05)	0.14
Gynecological	53	1.0 (Ref)	0.55 (0.27–1.13)	1.0 (Ref)	0.54 (0.22–1.32)	0.65 (0.25–1.64)	N/A	0.11
Colorectal	29	1.0 (Ref)	0.75 (0.29–1.91)	1.0 (Ref)	0.75 (0.25–2.26)	0.84 (0.23–3.10)	N/A	0.53
Hematological	30	1.0 (Ref)	0.83 (0.37–1.88)	1.0 (Ref)	1.13 (0.48–2.71)	0.37 (0.08–1.65)	1.10 (0.14–8.82)	0.40
Melanoma	25	1.0 (Ref)	5.13 (0.19–1.42)	1.0 (Ref)	0.86 (0.30–2.43)	0.18 (0.02–1.49)	N/A	0.07

§ Highest specific IgE scores recorded at baseline.

N/A = not applicable; n = number of participants; NMSC = nonmelanoma skin cancer; CNS = central nervous system.

All models were adjusted for sex (except for sex-specific analysis), socioeconomic status, period of measurement, history of chronic pulmonary disease and serum total IgE.

**Figure 1.**
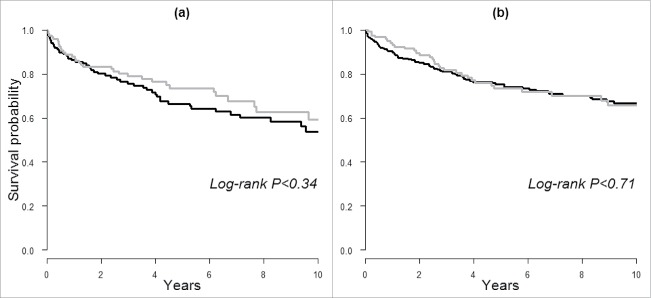
Kaplan–Meier curves of 10-y survival following cancer diagnosis in (A) men and (B) women based on prediagnostic IgE sensitization. Black lines indicate IgE sensitization and the gray lines indicate a lack thereof.

In our secondary analysis, we assessed risk of death following cancer diagnosis. As shown by the Kaplan–Meier curves in Fig. 1, the probability of survival was lower in men with IgE sensitization compared to those without in the long-term follow-up, but there were no statistically significant difference (Log-rank *p* > 0.05). Similarly, no differences were observed when categories of allergen-specific IgE scores were used (results not shown). We further evaluated this association by conducting Cox regression and found no clear associations between IgE sensitization or specific IgE scores and death from all-causes or cancer, e.g. HR for cancer death was 1.04 (95% CI: 0.59–1.84) and 1.54 (0.91–2.62) for men and women with compared to without IgE sensitization, respectively (results not shown in tables).

## Discussion

In the present study, IgE sensitization was associated with a lower risk of incident cancer. The inverse trend between allergen-specific IgE was more pronounced in women and among those with high total IgE levels. Among the most common cancers, no association was observed except for an inverse trend between serum specific IgE scores and risk of melanoma in the overall population, and with risk of breast and gynecological cancer in women. No associations between prediagnostic allergen-specific IgE and survival following cancer diagnosis were observed.

A shift toward immunosuppressive immune responses is characteristic of cancer.^22^ Nevertheless, little is known about the role of humoral immune responses, particularly IgE, in carcinogenesis. IgE production and class-switch recombination (CSR) to IgE from other immunoglobulin types such as IgG are regulated by T_H_2, and it has been suggested that a biased T_H_2 response underlies high IgE levels in allergic individuals.^23^ Since only limited responses following allergen exposure are observed for IgE,^24^ it is possible that any impact on carcinogenesis is secondary to the biased T_H_2 response rather than a result of high circulating IgE itself. In support of this, a temporal model of IgE and IgG has been proposed, in which the early-response IgE undergo sequential CSR to higher-affinity IgG3, then to IgG1, IgG2, and finally IgG4,^25-27^ the latter of which displays low immunoactivatory functions. Furthermore, inflammatory T_H_2-biased conditions such as IL-10, IL-4, VEGF and FoxP3^+^ Tregs that support class switching to IgG4 rather than IgE, and elevated IgG4 levels have been reported in different tumors including melanoma.^28,29^ Specifically in melanoma, elevated serum IgG4 levels and IgG4^+^ B cells in patient circulation are associated with worse clinical outcomes, implying a bias toward an alternative rather than an IgE-biased response associated with melanoma cancer growth.^30,31^ Taken together, these indications point toward a role of CSR dysregulation associated with T_H_2-biased response in driving the link between allergy, circulating IgE, and IgG4-related diseases including some cancers.^32^ Nevertheless, our findings in individuals with high total IgE (≥100 kU/L) may be consistent with a potential requirement for a critical threshold toward a classical IgE, rather than an alternative IgG4-biased T_H_2 immune response activation to confer for any potential protective benefits from cancer.

To date, only few prospective studies have examined possible associations between prediagnostic IgE sensitization and risk of incident cancer. In a recent study, Skaaby and colleagues evaluated serum specific evaluated IgE against inhalant allergen among 14,849 individuals.^11^ A lack of association between allergen-specific IgE levels and risk of overall cancer was reported for IgE sensitization.^11^ Similarly, we found a lack of a statistically significant association when assessing IgE sensitization against inhalant allergens in relation to overall incident cancer. However, when we took into account serum total IgE, an inverse association was found. Differences in follow-up periods and cohort composition may explain the discrepancy in the findings. Our study was based on a large cohort with a median follow-up of 18.6 y. In comparison, the study by Skaaby and colleagues comprised five cohorts spanning over different time periods, with a shorter overall median follow-up of 11.8 y.^11^ Adjustments for other risk factors such as smoking, alcohol consumption and physical activity did not alter findings in that study and are thus unlikely to explain the discrepancy with the results in our study in which risk factor information was unavailable.

For specific cancers, observational findings seem to vary by demographics and timing of specific IgE measurements. Two European nested case-control studies demonstrated an inverse association between IgE sensitization against inhalant allergens and risk of glioma in women but not men,^13,33^ whereas a lack of association was reported by another nested case-control study based on four US cohorts.^34^ Besides population attributes, a smaller number of cases in the latter study may explain this inconsistency. In case-control studies where allergen-specific IgE in cases was assessed after diagnosis, IgE sensitization was inversely associated with risk of lymphoid malignancies and positively with prostate cancer risk.^35,36^ However, no such association was observed in nested case-control studies where serum samples were prospectively collected before diagnosis.^11,12,35^

In our study population, we found no associations between IgE sensitization and risk of specific cancer sites, which is comparable with the study conducted by Skaaby and colleagues.^11^ However, an inverse association between serum specific IgE scores and risk of breast and gynecological cancers were observed in women. To date, evidence from observational studies on the role of allergy in these cancers remains unclear. In a meta-analysis, a lack of associations between history of any allergy, asthma or hay fever and breast cancer risk was suggested.^9^ On the other hand, a reduced incidence of ovarian, endometrial and cervical cancers have been reported in allergic patients.^37-40^ There is little evidence based on IgE sensitization status except for cancer of the uterus, where a lack of association was suggested.^11^ Considering the influence of estrogen in the development of these cancers, these results may indicate an interplay between immunologic and hormonal factors in carcinogenesis. Interestingly, endocrine treatment agents for estrogen-positive (ER+) breast cancers such as tamoxifen has been shown to reduce allergen-specific immunoglobulin levels including IgE in animal models of atopic dermatitis,^41^ which further suggests such complex associations.

There are several caveats in assessing serum allergen-specific IgE as a marker of allergy. Allergen-specific IgE levels represent the probability of having clinical allergic disease, therefore, use of a single allergen and/or cut-off to define IgE sensitization may not fully be representative of one's allergic symptoms.^42^ In line with this notion, we found stronger associations with categories of specific IgE compared to the conventional single cut-off point of specific IgE levels, which indicates that specific IgE scores or categories may be more useful than a single cut-off point in assessing cancer risk. Additionally, it was suggested that the number of positive inhalant allergens correlates better with allergic diseases compared to a single positive allergen-specific IgE test.^43^ In our study, we have addressed this possibility by an additional adjustment for the number of allergen tested, the number of positive results, or the ratio between the two, and they did not alter our findings. Finally, there is an indication that the sum of specific IgE levels against common inhalant allergens correlates better with clinical symptoms such as wheezing ^44^ and hospitalization with asthma,^45^ compared to individual levels of specific IgE. We were unable to assess cumulative levels of specific IgE in this study. Therefore, future studies assessing allergy-related cancer susceptibility may benefit from refined criteria of IgE sensitization.

To date, this is the first study documenting the association between IgE sensitization to common inhalant allergens and the risk of cancer using both serum allergen-specific and total IgE, and also the first study investigating the impact of pre-diagnostic IgE on cancer survival. Strengths of our study include the prospectively collected allergen-specific and total IgE levels prior to the diagnosis of cancer. Complete follow-up was obtained and all laboratory measurements were performed in one and the same laboratory.^14^ By using age as a timescale, we addressed the strong influence of age on absolute levels of specific IgE and its relative proportion to total IgE.^19,46^ A limitation of this study is the lack of information on clinical symptoms of allergy. Although information on specific types of allergens was available, we were unable to link individual allergens with risk of cancer due to the lack of number of cases. Our study population only included individuals who underwent IgE testing as part of a check-up or as outpatients and therefore may not be representative of the general population. However, this is not expected to influence the internal validity of this study. Allergy symptoms may have been confused with smoking-related respiratory disorders. To account for the lack of information on smoking, we adjusted our analysis for history of hospitalization with chronic obstructive pulmonary disease and asthma. Nevertheless, residual confounding may still have occurred. Lastly, spurious correlations may be of concern when performing multiple comparisons as shown in our study. However, we planned our analyses based on prior evidence and our results are explicable by suggested biological pathways and findings from other studies. Therefore, the observed association is unlikely to be spurious, although a discrepancy with the strength of the true association is possible due to the lack of cases.

In summary, our study suggests that IgE sensitization is weakly associated with a lower risk of malignancy in cancer-free individuals. These findings add to the evidence that immune responses involved in allergy contribute to the susceptibility of being diagnosed with cancer, particularly female breast and gynecological cancers and melanoma. In particular, our results may support a role of T_H_2-biased immune response in development of these cancers, indicated by a shift in the balance between circulating IgE and IgG subclasses including the low immunoactivatory IgG4, which urges further mechanistic investigations.

## Methods

### Study population

The AMORIS study has been described in detail elsewhere.^14^ Briefly, this study includes Swedish men and women with blood samples sequentially sent to the Central Automation Laboratory (CALAB) in Stockholm, Sweden^14^ Participants were either healthy and had a laboratory testing as a part of general health check-up, or were outpatients referred for laboratory testing. None of the participants were inpatients when samples were collected. In the AMORIS study, the CALAB database is linked to Swedish national registries, providing complete follow-up information on cancer diagnosis, death and emigration.^15^

Following a recent update, the AMORIS study now includes laboratory measurements of 812,073 individuals with follow-up information until 31 December 2011. From this population, we included 8,727 men and women aged 20 and older with no history of cancer who had baseline measurements of allergen-specific IgE against inhalant allergen and total IgE concentrations between 1992 and 1996. The study complied with the Declaration of Helsinki and was approved by the Ethics Review Board of the Karolinska Institutet.

### Assessment of outcome

Cancer diagnosis was obtained from the population-based Swedish Cancer Register. International Classification of Diseases, seventh revision (ICD-7) codes were used to classify cancer sites. In addition to overall cancer (ICD-7: 140–207), we assessed all cancer excluding non-melanoma skin cancer (NMSC) (ICD-7: 140–207 excluding 191) and the 10 most frequently diagnosed cancers in our study population: prostate (ICD-7: 177), female breast (ICD-7: 170), colorectal (ICD-7: 153–154), gynecological including ovarian, uterus and cervix (ICD-7: 171–176), hematological (ICD-7: 200–207), melanoma skin (ICD-7: 190), pulmonary (primary; ICD-7: 162), bladder (ICD-7: 181), NMSC (ICD-7: 191), central nervous system (CNS; ICD-7: 193) and kidney cancer (ICD-7: 180). The secondary outcomes of this study were all-cause and cancer-specific deaths. Dates and causes of death were obtained from the Swedish Cause of Death Register, whereas information on emigration was retrieved from the Migration Register.

### Assessment of exposures and covariates

Specific IgE concentrations against common inhalant allergens were measured using immunoassay. The test system, Pharmacia CAP^®^ System (Thermo Fisher Scientific, formerly Pharmacia Diagnostics AB, Uppsala, Sweden), is based on solid phase coupled allergen, adsorbing the IgE antibodies in the sample and assessed by an anti-IgE antibody commercially developed. It has been well standardized, shows correct quantitative values and is reproducible over time.^16^ A list of inhalant allergens tested is available in Table S1. Results of allergen-specific IgE test were expressed as scores ranging from 0 to 6 which represent different levels of IgE from undetectable up to high concentrations of IgE (kU/L) as displayed in Table 2. Apart from these scores, no information on continuous levels of specific IgE was available. As with previous studies, any scores higher than 0 (which correspond to specific IgE levels of ≥0.35 kU/L) were defined as IgE sensitization and the presence of atopy.^17^ For consistency, the term IgE sensitization was used to describe specific IgE levels ≥0.35 kU/L throughout this study. When multiple allergens were tested at baseline, results for all allergen-specific IgE measurements were collected and positive IgE sensitization was defined as having at least one positive result among all the tested allergens. In addition to IgE sensitization status, highest specific IgE scores recorded at baseline examinations when multiple allergens were tested were used in the analysis. Serum total IgE (kU/L) was measured by enzyme-linked immunosorbent assay (ELISA) using Immunoassay System ES 700 (Boehringer-Mannheim, Germany). Coefficient of variation was less than 5%. Total IgE levels were categorized based on the clinical cut-off points into low (<25 kU/L), moderate (25–100 kU/L) or high levels (≥100 kU/L) as previously described (173).

Age at baseline measurement (years) was collected from the CALAB database. The period of measurement was categorized (1992–1993, 1994–1995, 1996) to account for a long recruitment period. Socioeconomic status (SES; white collar, blue collar, unemployed or unknown) was based on national Censuses.^18^ From the National Patient Register, we used data on history of hospitalization with chronic pulmonary disease including asthma (ever, never).

### Statistical analysis

Multivariable Cox regression was used to estimate HR with corresponding 95% CI of overall risk of cancer by IgE sensitization status (yes, no) in all participants. Follow-up time was defined as the time from baseline measurement until cancer diagnosis, death from any cause, emigration or end of study, whichever occurred first. Additionally, the trend between allergen-specific IgE scores against inhalant allergens and risk of cancer was evaluated by assessing scores in groups (0, 1–2, 3–4, 5–6) as an ordinal scale. Levels of allergen-specific IgE are known to substantially decrease with age.^19^ Therefore, all analyses were performed using age as the time scale with delayed entry.

Models were adjusted for sex, SES, and period of measurement, history of chronic pulmonary disease to account for asthma and as a proxy for smoking given their association to IgE sensitivity and risk of lung cancer.^20,21^ Analyses were repeated in men and women separately. A further adjustment for categories of total IgE levels was performed in the second model. To evaluate any effect modification by total IgE levels, analyses were stratified according to total IgE levels. In an additional analysis, we adjusted our model for the number of allergen tested, the number of positive results or the ratio between the two. Analysis was subsequently performed for all cancer excluding NMSC and the 10 most common cancer sites with adjustment for total IgE levels. We repeated a similar analysis in men and women but only assessed the five sex-specific most common cancer sites due to the lack of number of cases.

For our secondary objective, we studied pre-diagnostic allergen-specific IgE in relation to survival after cancer diagnosis. Three cancer patients were excluded in the analysis because the diagnosis of cancer occurred at the time of death, leaving 686 individuals with cancer in the final analysis. Follow-up time was defined as the time from cancer diagnosis until death from any cause, emigration or end of study, whichever occurred first. Kaplan–Meier curves were used to assess overall survival by IgE sensitization status and scores of allergen-specific IgE, and statistical differences were assessed with the log-rank test. Cox regression was used to quantify the risks of all-cause and cancer-specific deaths by IgE sensitization status and allergen-specific IgE scores with age at diagnosis as time scale. The models were adjusted for the interval between baseline IgE measurements and cancer diagnosis, and total IgE levels.

All analyses were conducted with Statistical Analysis Software (SAS) release 9.4 (SAS Institute, Cary, NC) and R version 3.0.2 (R Foundation for Statistical Computing).

## Supplementary Material

KONI_A_1154250_s02.docx
