# Supplementary material for: Investigating the association between allergen-specific immunoglobulin E, cancer risk and survival
Source: Oncoimmunology. 2016 Mar 28;5(6):e1154250. doi: 10.1080/2162402X.2016.1154250 (PMC4938379; doi:10.1080/2162402X.2016.1154250)
Supplement: KONI_A_1154250_s02.docx [file koni-05-06-1154250-s001.docx]

**Supplementary Data**

**Table S1**. List of individual inhalant allergens tested in the cohort

| **CAP code** | **Description** |
| --- | --- |
| D1 | House dust: *Dermatophagoides pteronyssinus* IgE |
| D2 | House dust:  *Dermatophagoides farinae* IgE |
| D3 | Mites: *Dermamophagoides microceras* IgE |
| D70 | Mites:  *Acarus siro* IgE |
| D71 | Mites:  *Lepidoglyphus destructor* IgE |
| D72 | Mites:  *Tyrophagus putresentiae* IgE |
| D73 | Mites:  *Glycyphagus domesticus* IgE |
| D74 | Mites:  *Euroglyphus maynei* IgE |
| E1 | Cat dander IgE |
| E3 | Horse dander IgE |
| E4 | Cow dander IgE |
| E5 | Dog dander IgE |
| E6 | Guinea pig epithelium IgE |
| E70 | Goose feathers IgE |
| E78 | Parrot (budgerigar) feathers IgE |
| E82 | Rabbit epithelium IgE |
| E84 | Hamster epithelium IgE |
| E85 | Chicken feathers IgE |
| E86 | Duck feathers IgE |
| E87 | Rat IgE |
| E89 | Turkey feathers IgE |
| G1 | Grass pollen: Sweet vernal IgE |
| G12 | Grass pollen: Cultivated rye EgE |
| G13 | Grass pollen: Velvet grass IgE |
| G5 | Grass pollen: Perennial rye-grass IgE |
| G6 | Grass pollen: Timothy grass IgE |
| G7 | Grass pollen: Common reed IgE |
| H1 | House dust IgE |
| H2 | House dust, Hollister-Stier Labs IgE |
| M1 | Molds/yeast: *Penicillium notatum* IgE |
| M2 | Molds/yeast: *Cladosporium herbarum* IgE |
| M3 | Molds/yeast: *Aspergillus fumigatus* IgE |
| M6 | Molds/yeast: *Alternaria tenuis* IgE |
| T12 | Tree pollen: Willow IgE |
| T14 | Tree pollen: Cottonwood IgE |
| T2 | Tree pollen: Grey alder IgE |
| T3 | Tree pollen: Common silver birch IgE |
| T4 | Tree pollen: Hazel IgE |
| T7 | Tree pollen: Oak IgE |
| T8 | Tree pollen: Elm IgE |
| T9 | Tree pollen: Olive IgE |
| W10 | Weed/flower pollen: Lambs quarters, Goosefoot IgE |
| W12 | Weed/flower pollen: Golden rod IgE |
| W19 | Weed/flower pollen: Wall Pellitory (*P. Officinalis*) IgE |
| W20 | Weed/flower pollen: Nettle IgE |
| W6 | Weed/flower pollen: Mugwort IgE |
| W7 | Weed/flower pollen: Marguerite, ox-eye daisy IgE |
| W8 | Weed/flower pollen: Dandelion IgE |
| W9 | Weed/flower pollen: English plantain, ribwort IgE |

**Total IgE (kU/L)**

**Figure S1**. Distribution of participants by serum specific and total IgE categories
